# Talking about menopause in the workplace

**DOI:** 10.1016/j.crwh.2021.e00306

**Published:** 2021-03-11

**Authors:** Sarah Carter, Ollie Jay, Kirsten I. Black

**Affiliations:** a University of Sydney, Thermal Ergonomics Laboratory, Sydney School of Health Sciences, Faculty of Medicine and Health, Sydney, Australia; b Sydney School of Medicine (Central Clinical School), Faculty of Medicine and Health, University of Sydney, Sydney, Australia

**Keywords:** Menopause, Workplace, health, and safety, Physiology, Heat stress, Hot flushes

Modernisation of the workforce, together with an ageing population, has seen increased participation rates of middle-aged and older women in industrialised countries [1]. With governments encouraging retention of older women in the workforce and the average age of retirement rising, women can be spending over a third of their working life after the menopause. The physiological and psychological changes women experience, particularly in the menopause transition, are well documented. There is now emerging interest in investigating how menopausal symptoms are experienced in the workplace [2], impact on workplace productivity [[2], [3], [4]], are affected by workplace stressors [5,6], as well as interest in the level of managerial support women in the menopause are provided in the workplace [6]. Whilst all symptoms of the menopause can be unsettling, the hot flush remains the most pervasive and inescapable symptom of oestrogen deficiency.

Researchers have called for menopause to be considered as a work, health, and safety (WHS) issue in an effort to retain female workers by supporting them through the menopause transition [[5], [6], [7]]. However, whilst the psychological effects on WHS are well characterised, the physical and physiological manifestations are yet to be meaningfully linked to workplace safety. Claims of an increased heat stress risk at work for menopausal women have not established a feasible connection between hot flush physiology and a heat stress response. In fact, to date, no physiological data have provided evidence that menopausal women are unable to effectively regulate their internal body temperature with environmental heat exposure. Nevertheless, the hot flush has potential to impact female workers' health and safety through other avenues. The focus of WHS policy pertaining to symptomatic menopausal women should instead be placed on the ripple effects, specifically, the acute and chronic effects of hot flush occurrences on menopausal women within and outside of the workplace.

The hot flush usually begins as a sudden hot sensation that spreads from the chest to the neck, head, and arms. Physiologically, the flush begins with significant increases to peripheral skin blood flow, sometimes accompanied by an increased heart rate and/or followed by localised sweating of the upper body. Normally, an increased heart rate accompanies increased skin blood flow requirements to ensure adequate cardiac output for the maintenance of blood pressure. The potential acute effects of a hot flush on heart rate may be further aggravated with increased physical work and/or environmental heat exposure as seen in occupational heat stress profiles [8]. As oestrogen is known to be cardioprotective through the vascular mechanisms it induces [9], losing a dominant oestrogen presence together with physical work or hot environments could further exacerbate the known risks cardiovascular disease poses to postmenopausal women. Further, maintenance of adequate hydration may also be an issue for menopausal women. As oestrogen plays a regulatory role in the cardiovascular system, so too is it intrinsically linked with fluid regulation. Studies have shown menopausal women have a reduced thirst sensitivity to changes in central body fluid volume, potentially resulting in dehydration from a slower rate of fluid replenishment [10]. Dehydration in respect of the previously highlighted conditions (arduous work and environmental conditions) could again further exacerbate cardiovascular strain in menopausal women.

Another acute effect of the hot flush could arise from the increased peripheral skin blood flow response, directly impacting cognitive performance. Unpleasant warmth can reportedly impact complex cognitive performance, affecting people's ability to concentrate and complete complex cognitive tasks when they perceive themselves to be uncomfortably hot [11,12]. In this instance, increased displeasure with the immediate environment could occur through either a rapid rate of change in local skin temperature or increased perception of heat [11,13]. It should be noted that the magnitude of skin temperature changes during hot flushes is inconsistent within the literature [[14], [15], [16]]. Whilst there may not always be large increases in local skin temperature, it is known that the rate of change, rather than the magnitude, can greatly influence thermal sensation [13,17]. In this respect, hot flushes through acute and rapid changes to skin blood flow could transiently decrease one's ability to perform work-related tasks which involve complex cognitive processes.

Women experiencing night sweats have their normal sleep-wake cycles acutely disrupted, sometimes with chronic consequences. The neurobiological processes which govern sleep can become dysregulated by consistent waking and sleep deprivation, such that decrements in cognitive performance can occur when awake [18]. Sleep deprivation can elicit sensitivity to waking, ultimately increasing the likelihood of more sleep disturbances. Whilst rest will remedy sleep deprivation, the cumulative effect of continued sleep disturbances will continue as long as the sleep cycles remain broken [18,19]. As a result, acute mental fatigue from continued awakenings and sleep deprivation can occur. Importantly, mental fatigue can increase the time spent on tasks, deteriorate performance [20], and reduce one's tolerance for exercise or physical activity through an increased perception of the workload performed [21]. Therefore, within a workplace, the latent impact of night sweats can manifest as reduced work capacity and productivity, with increased error occurrence, which could result in greater workplace accidents or injury rates [18,19].

The workplace can also impact upon the experience of menopausal symptoms; factors such as employment status or loss of pay can contribute to the frequency of hot flush occurrences in menopausal women [5,6]. The aforementioned physiological ramifications of the hot flush have the potential to impede employment status through reduced work output and absentia [3,4]. A vicious cycle can occur where emotional distress, financial standing, workload, and the social environment of a workplace can all contribute to stress, resulting in increased mental fatigue and hot flush occurrences [5,19]. Whilst workplace stressors can influence the hot flush frequency, the reverse paradigm is hot flush frequency can also exacerbate stress, cause emotional distress, and increase intentions to leave the workforce [2,6,7].

In conclusion, the retention of menopausal female workers is important to ensure age diversification and expertise remains within the modern workforce. For older women, menopause and hot flushes represent a potential occupational safety issue. Employers should consider menopausal symptoms within physiological contexts, rather than solely within a comfort framework, to emphasise the importance of female worker health. The experience of menopause in the workplace would benefit from research further exploring the physiological mechanisms of hot flushes, the thermal comfort requirements of women, and practical mitigation strategies to offset the negative effects associated with hot flushes. The objective of such research should be to enhance the ability of women in the menopause transition and beyond to optimise their workplace performance.
